# Supplementary material for: Difference in predictors and barriers to arts and cultural engagement with age in the United States: A cross-sectional analysis using the Health and Retirement Study
Source: PLoS One. 2021 Dec 20;16(12):e0261532. doi: 10.1371/journal.pone.0261532 (PMC8687585; doi:10.1371/journal.pone.0261532)
Supplement: S1 Table — (DOCX) [file pone.0261532.s001.docx]

***Supplementary Table S1 Missing data across arts outcomes***

|  | **Frequency of arts participation** | | | | **Difficulty participating in the arts** | | | | **Cultural event attendance** | | | | **Missed cultural events** | | | |  |
| --- | --- | --- | --- | --- | --- | --- | --- | --- | --- | --- | --- | --- | --- | --- | --- | --- | --- |
|  |  |  |  |  |  |  |  |  |  |  |  |  |  |  |  |  |  |
|  |  | | | | **N=1465** | | | | | | | | | | | |  |
|  | **< Weekly** | | **Weekly** | | **Disagree / neutral** | | **Agree** | | **No** | | **Yes** | | **No** | | **Yes** | |  |
|  | **N** | **%** | **N** | **%** | **N** | **%** | **N** | **%** | **N** | **%** | **N** | **%** | **N** | **%** | **N** | **%** |  |
| **Age three categories** |  |  |  |  |  |  |  |  |  |  |  |  |  |  |  |  |  |
| 50-59 | 242 | 27.3 | 1585 | 23.9 |  |  |  |  |  |  |  |  |  |  |  |  |  |
| 60-69 | 273 | 30.9 | 2043 | 30.8 |  |  |  |  |  |  |  |  |  |  |  |  |  |
| 70+ | 370 | 41.8 | 3010 | 45.3 |  |  |  |  |  |  |  |  |  |  |  |  |  |
| mi | **0** | **0** | **0** | **0** |  |  |  |  |  |  |  |  |  |  |  |  |  |
| **Age binary** |  |  |  |  |  |  |  |  |  |  |  |  |  |  |  |  |  |
| 50-69 |  |  |  |  | 545 | 60.4 | 317 | 56.3 | 267 | 50.8 | 595 | 63.4 | 504 | 54.0 | 358 | 67.3 |  |
| 70+ |  |  |  |  | 357 | 39.6 | 246 | 43.7 | 259 | 49.2 | 344 | 36.6 | 429 | 46.0 | 174 | 32.7 |  |
| mi |  |  |  |  | **0** | **0** | **0** | **0** | **0** | **0** | **0** | **0** | **0** | **0** | **0** | **0** |  |
| **Gender** |  |  |  |  |  |  |  |  |  |  |  |  |  |  |  |  |  |
| Female | 429 | 48.5 | 4060 | 61.2 | 548 | 60.8 | 349 | 62.0 | 307 | 58.4 | 590 | 62.8 | 541 | 58.0 | 356 | 66.9 |  |
| mi |  |  |  |  |  |  |  |  |  |  |  |  |  |  |  |  |  |
| **Ethnicity** |  |  |  |  |  |  |  |  |  |  |  |  |  |  |  |  |  |
| White | 562 | 63.5 | 5096 | 76.8 | 643 | 71.3 | 406 | 72.1 | 347 | 66.0 | 702 | 74.8 | 666 | 71.4 | 383 | 72.0 |  |
| Black/African American | 211 | 23.8 | 1069 | 16.1 | 189 | 21.0 | 103 | 18.3 | 127 | 24.1 | 165 | 17.6 | 185 | 19.8 | 107 | 20.1 |  |
| Other ethnicity [including American Indian  or Alaskan Native, Asian or Pacific Islander] | 112 | 12.7 | 470 | 7.1 | 70 | 7.8 | 54 | 9.6 | 52 | 9.9 | 72 | 7.7 | 82 | 8.8 | 42 | 7.9 |  |
| mi | 0 | 0.0 | 3 | 0.1 | **0** | **0** | **0** | **0** | **0** | **0** | **0** | **0** | **0** | **0** | **0** | **0** |  |
| **Marital status** |  |  |  |  |  |  |  |  |  |  |  |  |  |  |  |  |  |
| Married | 479 | 54.1 | 3959 | 59.6 | 527 | 58.4 | 296 | 52.6 | 245 | 46.6 | 578 | 61.6 | 531 | 56.9 | 292 | 54.9 |  |
| mi | 1 | 0.1 | 1 | 0.0 | 0 | 0.0 | 1 | 0.2 | 1 | 0.2 | 0 | 0.0 | 0 | 0.0 | 1 | 0.2 |  |
| **Educational attainment** |  |  |  |  |  |  |  |  |  |  |  |  |  |  |  |  |  |
| None | 289 | 32.7 | 894 | 13.5 | 152 | 16.9 | 108 | 19.2 | 169 | 32.1 | 91 | 9.7 | 200 | 21.4 | 60 | 11.3 |  |
| High School/ GED | 485 | 54.8 | 3544 | 53.4 | 436 | 48.3 | 317 | 56.3 | 285 | 54.2 | 468 | 49.8 | 483 | 51.8 | 270 | 50.8 |  |
| College / postgraduate | 101 | 11.4 | 2143 | 32.3 | 303 | 33.6 | 131 | 23.3 | 70 | 13.3 | 364 | 38.8 | 244 | 26.2 | 190 | 35.7 |  |
| mi | 10 | 1.1 | 57 | 0.9 | 11 | 1.2 | 7 | 1.2 | 2 | 0.4 | 16 | 1.7 | 6 | 0.6 | 12 | 2.3 |  |
| **Neighbourhood safety** |  |  |  |  |  |  |  |  |  |  |  |  |  |  |  |  |  |
| Fair/Poor | 159 | 18.0 | 664 | 10.0 | 82 | 9.1 | 83 | 14.7 | 94 | 17.9 | 71 | 7.6 | 100 | 10.7 | 65 | 12.2 |  |
| mi | 29 | 3.3 | 78 | 1.2 | 14 | 1.6 | 12 | 2.1 | 14 | 2.7 | 12 | 1.3 | 13 | 1.4 | 13 | 2.4 |  |
| **Employment status** |  |  |  |  |  |  |  |  |  |  |  |  |  |  |  |  |  |
| Employed | 235 | 26.6 | 2125 | 32.0 | 152 | 16.9 | 108 | 19.2 | 169 | 32.1 | 91 | 9.7 | 200 | 41.4 | 60 | 11.3 |  |
| Unemployed/ inactive | 268 | 30.3 | 1183 | 17.8 | 436 | 48.3 | 317 | 56.3 | 285 | 54.2 | 468 | 49.8 | 483 | 51.8 | 270 | 50.8 |  |
| Retired | 374 | 42.3 | 3280 | 49.4 | 303 | 33.6 | 131 | 23.3 | 70 | 13.3 | 364 | 38.8 | 244 | 26.2 | 190 | 35.7 |  |
| mi | 8 | 0.9 | 50 | 0.8 | **11.0** | **1.2** | **7.0** | **1.2** | 2 | 0.4 | 16 | 1.7 | **6.0** | **0.6** | **12.0** | **2.3** |  |
| **Wealth, quartiled** |  |  |  |  |  |  |  |  |  |  |  |  |  |  |  |  |  |
| Quartile 1 | 339 | 38.3 | 1293 | 19.5 | 185 | 20.5 | 167 | 29.7 | 193 | 36.7 | 159 | 16.9 | 218 | 23.4 | 134 | 25.2 |  |
| Quartile 2 | 262 | 29.6 | 1530 | 23.1 | 221 | 24.5 | 153 | 27.2 | 146 | 27.8 | 228 | 24.3 | 232 | 24.9 | 142 | 26.7 |  |
| Quartile 3 | 171 | 19.3 | 1814 | 27.3 | 217 | 24.1 | 128 | 22.7 | 113 | 21.5 | 232 | 24.7 | 230 | 24.7 | 115 | 21.6 |  |
| Quartile 4 | 113 | 12.8 | 2001 | 30.1 | 279 | 30.9 | 115 | 20.4 | 74 | 14.1 | 320 | 34.1 | 253 | 27.1 | 141 | 26.5 |  |
| mi | **0** | **0** | **0** | **0** | **0** | **0** | **0** | **0** | **0** | **0** | **0** | **0** | **0** | **0** | **0** | **0** |  |
| **Satisfied with aging** |  |  |  |  |  |  |  |  |  |  |  |  |  |  |  |  |  |
| Yes | 612 | 69.2 | 5495 | 82.8 | 282 | 31.3 | 173 | 30.7 | 150 | 28.5 | 305 | 32.5 | 284 | 30.4 | 171 | 32.1 |  |
| mi | 25 | 2.8 | 70 | 1.1 | 538 | 59.7 | 325 | 57.7 | 316 | 60.1 | 547 | 58.3 | 570 | 61.1 | 293 | 55.1 |  |
| **Satisfied with Life** |  |  |  |  |  |  |  |  |  |  |  |  |  |  |  |  |  |
| Yes | 585 | 66.1 | 5103 | 76.9 | 309 | 34.3 | 176 | 31.3 | 162 | 30.8 | 323 | 34.4 | 298 | 31.9 | 187 | 35.2 |  |
| mi | 20 | 2.3 | 62 | 0.9 | 541 | 60.0 | 330 | 58.6 | 321 | 61.0 | 550 | 58.6 | 58 | 6.2 | 51 | 9.6 |  |
| **Attend religious services** |  |  |  |  |  |  |  |  |  |  |  |  |  |  |  |  |  |
| None | 166 | 18.8 | 751 | 11.3 | 237 | 26.3 | 154 | 27.4 | 40 | 7.6 | 39 | 4.2 | 254 | 27.2 | 137 | 25.8 |  |
| Monthly/less | 331 | 37.4 | 2668 | 40.2 | 299 | 33.2 | 201 | 35.7 | 78 | 14.8 | 174 | 18.5 | 314 | 33.7 | 186 | 35.0 |  |
| Weekly | 207 | 23.4 | 2552 | 38.5 | 361 | 40.0 | 206 | 36.6 | 59 | 11.2 | 153 | 16.3 | 362 | 38.8 | 205 | 38.5 |  |
| mi | 181 | 20.5 | 667 | 10.1 | 5 | 0.6 | 2 | 0.4 | 349 | 66.4 | 573 | 61.0 | 3 | 0.3 | 4 | 0.8 |  |
| **See friends** |  |  |  |  |  |  |  |  |  |  |  |  |  |  |  |  |  |
| Yearly/less | 299 | 33.8 | 1735 | 26.1 | 50 | 5.5 | 29 | 5.2 | 181 | 34.4 | 210 | 22.4 | 61 | 6.5 | 18 | 3.4 |  |
| Monthly | 308 | 34.8 | 2128 | 32.1 | 161 | 17.9 | 91 | 16.2 | 162 | 30.8 | 338 | 36.0 | 138 | 14.8 | 114 | 21.4 |  |
| Weekly | 275 | 31.1 | 2755 | 41.5 | 129 | 14.3 | 83 | 14.7 | 181 | 34.4 | 386 | 41.1 | 127 | 13.6 | 85 | 16.0 |  |
| mi | 3 | 0.3 | 20 | 0.3 | 562 | 62.3 | 360 | 63.9 | 2 | 0.4 | 5 | 0.5 | 607 | 65.1 | 315 | 59.2 |  |
| **Depression CES-D** |  |  |  |  |  |  |  |  |  |  |  |  |  |  |  |  |  |
| Present | 200 | 22.6 | 839 | 12.6 | 104 | 11.5 | 117 | 20.8 | 122 | 23.2 | 99 | 10.5 | 115 | 12.3 | 106 | 19.9 |  |
| mi | 73 | 8.3 | 128 | 1.9 | **0** | **0** | **0** | **0** | **0** | **0** | **0** | **0** | **0** | **0** | **0** | **0** |  |
| **Smoker** |  |  |  |  |  |  |  |  |  |  |  |  |  |  |  |  |  |
| Yes | 159 | 18.0 | 744 | 11.2 | 108 | 12.0 | 89 | 15.8 | 102 | 19.4 | 95 | 10.1 | 120 | 12.9 | 77 | 14.5 |  |
| mi | 352 | 39.8 | 3102 | 46.7 | 430 | 47.7 | 252 | 44.8 | 218 | 41.4 | 464 | 49.4 | 430 | 46.1 | 252 | 47.4 |  |
| **Self-rated health** |  |  |  |  |  |  |  |  |  |  |  |  |  |  |  |  |  |
| Fair/Poor | 421 | 47.6 | 1657 | 25.0 | 204 | 22.6 | 209 | 37.1 | 229 | 43.5 | 184 | 19.6 | 252 | 27.0 | 161 | 30.3 |  |
| mi | 1 | 0.1 | 4 | 0.1 | 1 | 0.1 | 0 | 0.0 | 0 | 0.0 | 1 | 0.1 | 1 | 0.1 | 0 | 0.0 |  |
| **iADL** |  |  |  |  |  |  |  |  |  |  |  |  |  |  |  |  |  |
| None | 514 | 58.1 | 5262 | 79.3 | 740 | 82.0 | 404 | 71.8 | 349 | 66.4 | 795 | 84.7 | 736 | 78.9 | 408 | 76.7 |  |
| Difficulties with activities | 333 | 37.6 | 1268 | 19.1 | 145 | 16.1 | 144 | 25.6 | 158 | 30.0 | 131 | 14.0 | 175 | 18.8 | 114 | 21.4 |  |
| Unable to do activities | 38 | 4.3 | 107 | 1.6 | 17 | 1.9 | 15 | 2.7 | 19 | 3.6 | 13 | 1.4 | 22 | 2.4 | 10 | 1.9 |  |
| mi | 0 | 0.0 | 1 | 0.0 | **0** | **0** | **0** | **0** | **0** | **0** | **0** | **0** | **0** | **0** | **0** | **0** |  |
| **Long term conditions** |  |  |  |  |  |  |  |  |  |  |  |  |  |  |  |  |  |
| Yes | 619 | 69.9 | 4266 | 64.3 | 561 | 62.2 | 373 | 66.3 | 374 | 71.1 | 560 | 59.6 | 347 | 37.2 | 184 | 34.6 |  |
| mi | **0** | **0** | **0** | **0** | **0** | **0** | **0** | **0** | **0** | **0** | **0** | **0** | 586 | 62.8 | 348 | 65.4 |  |
| **Cognition score, quartiled** |  |  |  |  |  |  |  |  |  |  |  |  |  |  |  |  |  |
| Quartile 1 | 302 | 34.1 | 1,494 | 22.6 | 171 | 24.2 | 162 | 28.8 | 208 | 39.5 | 167 | 17.8 | 275 | 29.5 | 100 | 18.8 |  |
| Quartile 2 | 292 | 33.0 | 2,234 | 33.7 | 197 | 27.9 | 204 | 36.2 | 187 | 36.0 | 283 | 30. | 298 | 31.9 | 172 | 32.3 |  |
| Quartile 3 | 138 | 16.9 | 1,401 | 21.1 | 171 | 24.2 | 99 | 17.6 | 74 | 14.1 | 235 | 25.0 | 194 | 20.8 | 115 | 21.6 |  |
| Quartile 4 | 80 | 9.0 | 1,377 | 20.7 | 167 | 23.7 | 98 | 17.4 | 57 | 10.8 | 254 | 27.1 | 166 | 19.8 | 145 | 27.3 |  |
| mi | 73 | 8.3 | 128 | 1.93 | 0 | 0 | 0 | 0 | 0 | 0 | 0 | 0 | 0 | 0 | 0 | 0 |  |
| **Arts index** |  |  |  |  |  |  |  |  |  |  |  |  |  |  |  |  |  |
| mi | 817 | 92.3 | 6090 | 91.7 | **0** | **0** | **0** | **0** | **0** | **0** | **0** | **0** | **0** | **0** | **0** | **0** |  |

Note. ‘mi’ indicates missing data for each predictor variable
