# Supplementary material for: Difference in predictors and barriers to arts and cultural engagement with age in the United States: A cross-sectional analysis using the Health and Retirement Study
Source: PLoS One. 2021 Dec 20;16(12):e0261532. doi: 10.1371/journal.pone.0261532 (PMC8687585; doi:10.1371/journal.pone.0261532)
Supplement: S2 Table — (DOCX) [file pone.0261532.s002.docx]

***Supplementary Table S2. Age related differences in predictors of frequency of arts participation- two age categories from logistic regression models***

|  |  | | | | | | | |  |
| --- | --- | --- | --- | --- | --- | --- | --- | --- | --- |
|  |  |  |  |  |  |  |  |  |  |
|  | **Ages 50-69^A^** | | | | **Ages ≥ 70 ^B^** | | | |  |
|  | **N= 4050** | | | | **N= 3244** | | | |  |
|  | ***OR*** | ***95%CI*** | | ***P*** | ***OR*** | ***95%CI*** | | ***P*** |  |
| **Gender (Ref Female)** | -- |  |  |  |  |  |  |  |  |
| Male | **0.52** | **0.38** | **0.72** | **<0.001** | **0.44** | **0.31** | **0.64** | **<0.001** |  |
| **Ethnicity (Ref White)** | -- |  |  |  |  |  |  |  |  |
| African American | 1.01 | 0.68 | 1.50 | 0.963 | 0.72 | 0.46 | 1.15 | 0.168 |  |
| Other ethnicity [including American Indian or  Alaskan Native, Asian or Pacific Islander] | 0.98 | 0.60 | 1.61 | 0.929 | 0.51 | 0.28 | 0.92 | 0.026 |  |
| **Marital status (Ref Married)** | -- |  |  |  |  |  |  |  |  |
| Unmarried | 1.05 | 0.76 | 1.46 | 0.761 | 0.92 | 0.63 | 1.35 | 0.673 |  |
| **Educational attainment (Ref None)** | -- |  |  |  |  |  |  |  |  |
| High School/ GED | 1.39 | 0.90 | 2.14 | 0.135 | **2.30** | **1.57** | **3.35** | **<0.001** |  |
| College / postgraduate | **2.24** | **1.28** | **3.92** | **0.005** | **6.04** | **3.21** | **11.37** | **<0.001** |  |
| **Neighbourhood safety (Ref Good/excellent)** | -- |  |  |  |  |  |  |  |  |
| Fair/Poor | 1.12 | 0.73 | 1.70 | 0.602 | 1.24 | 0.77 | 1.98 | 0.374 |  |
| **Employment status (Ref Employed)** | -- |  |  |  |  |  |  |  |  |
| Unemployed/ Inactive | 0.98 | 0.67 | 1.44 | 0.922 | 0.51 | 0.23 | 1.11 | 0.088 |  |
| Retired | 1.21 | 0.85 | 1.72 | 0.297 | 0.59 | 0.29 | 1.20 | 0.145 |  |
| **Wealth, quartiled (Ref Quartile 1)** | -- |  |  |  |  |  |  |  |  |
| Quartile 2 | **1.72** | **1.17** | **2.53** | **0.006** | 1.10 | 0.73 | 1.66 | 0.649 |  |
| Quartile 3 | **1.73** | **1.10** | **2.72** | **0.017** | 1.23 | 0.79 | 1.93 | 0.360 |  |
| Quartile 4 | **1.94** | **1.13** | **3.33** | **0.017** | 1.53 | 0.88 | 2.68 | 0.135 |  |
| **Satisfied with aging (Ref Yes)** | -- |  |  |  |  |  |  |  |  |
| No | 1.36 | 0.93 | 1.99 | 0.113 | **1.66** | **1.11** | **2.48** | **0.013** |  |
| **Satisfied with Life (Ref Yes)** | -- |  |  |  |  |  |  |  |  |
| No | 0.90 | 0.64 | 1.28 | 0.565 | 1.18 | 0.80 | 1.73 | 0.400 |  |
| **See friends (Ref Yearly/less)** | -- |  |  |  |  |  |  |  |  |
| Monthly | 1.06 | 0.70 | 1.62 | 0.776 | 1.17 | 0.74 | 1.84 | 0.507 |  |
| Weekly | **1.66** | **1.04** | **2.65** | **0.032** | 1.41 | 0.88 | 2.27 | 0.151 |  |
| **Attend religious services (Ref Yearly/Less)** | -- |  |  |  |  |  |  |  |  |
| Monthly | 1.15 | 0.81 | 1.64 | 0.435 | 0.68 | 0.45 | 1.02 | 0.064 |  |
| Weekly | 1.14 | 0.77 | 1.68 | 0.517 | 1.47 | 0.97 | 2.21 | 0.070 |  |
| **Depression CES-D (Ref None)** | -- |  |  |  |  |  |  |  |  |
| Present | 0.87 | 0.57 | 1.32 | 0.515 | 0.75 | 0.49 | 1.16 | 0.197 |  |
| **Smoker** | -- |  |  |  |  |  |  |  |  |
| Yes | 0.87 | 0.56 | 1.37 | 0.553 | 1.56 | 0.84 | 2.89 | 0.159 |  |
| **Self-rated health (Ref Good/excellent)** | -- |  |  |  |  |  |  |  |  |
| Fair/Poor | 1.20 | 0.82 | 1.75 | 0.354 | 0.93 | 0.65 | 1.32 | 0.680 |  |
| **iADL (Ref None)** | -- |  |  |  |  |  |  |  |  |
| Difficulties with activities | 0.79 | 0.54 | 1.17 | 0.236 | **0.64** | **0.45** | **0.91** | **0.012** |  |
| Unable to do activities | **0.33** | **0.13** | **0.80** | **0.014** | **0.31** | **0.14** | **0.67** | **0.003** |  |
| **Long term conditions (Ref None)** | -- |  |  |  |  |  |  |  |  |
| Yes | 0.92 | 0.67 | 1.26 | 0.586 | 0.79 | 0.51 | 1.22 | 0.284 |  |
| **Total cognition score, quartiled (Ref Quartile 1)** | -- |  |  |  |  |  |  |  |  |
| Quartile 2 | 1.35 | 0.88 | 2.06 | 0.170 | 1.03 | 0.72 | 1.48 | 0.861 |  |
| Quartile 3 | 1.23 | 0.75 | 2.02 | 0.421 | 1.03 | 0.59 | 1.83 | 0.910 |  |
| Quartile 4 | 1.67 | 0.97 | 2.88 | 0.064 | **2.88** | **1.24** | **6.66** | **0.014** |  |
| **Arts index** | **0.62** | **0.42** | **0.92** | **0.017** | **0.65** | **0.45** | **0.94** | **0.024** |  |

Note. Dashes indicate reference category.

Frequency of participation is: Weekly or more vs Less than weekly to participants ‘How often do you [do writing, bake or cook, sew or knit, read, do hobbies, participate in a community arts group]’

Columns A-B show age stratified analyses
