# Supplementary material for: Difference in predictors and barriers to arts and cultural engagement with age in the United States: A cross-sectional analysis using the Health and Retirement Study
Source: PLoS One. 2021 Dec 20;16(12):e0261532. doi: 10.1371/journal.pone.0261532 (PMC8687585; doi:10.1371/journal.pone.0261532)
Supplement: S3 Table — (DOCX) [file pone.0261532.s003.docx]

***Supplementary Table S3. Age related differences in reported difficulties participating in the arts- neutral/agree vs disagree from logistic regression models***

|  |  | | | | | | | |  |
| --- | --- | --- | --- | --- | --- | --- | --- | --- | --- |
|  |  |  |  |  |  |  |  |  |  |
|  | **Ages 50-69^A^** | | | | **Ages ≥ 70^B^** | | | |  |
|  | **N= 862** | | | | **N= 603** | | | |  |
|  | *OR* | *95%CI* | | *P* | *OR* | *95%CI* | | *P* |  |
| **Gender (Ref Female)** | -- |  |  |  |  |  |  |  |  |
| Male | 1.35 | 0.89 | 2.07 | 0.163 | 0.76 | 0.48 | 1.21 | 0.249 |  |
| **Ethnicity (Ref White)** | -- |  |  |  |  |  |  |  |  |
| African American | **0.51** | **0.29** | **0.88** | **0.016** | **0.54** | **0.29** | **1.00** | **0.048** |  |
| Other ethnicity [including American Indian or  Alaskan Native, Asian or Pacific Islander] | 0.96 | 0.48 | 1.93 | 0.909 | 0.57 | 0.18 | 1.78 | 0.329 |  |
| **Marital status (Ref Married)** | -- |  |  |  |  |  |  |  |  |
| Unmarried | 1.03 | 0.65 | 1.63 | 0.886 | 1.06 | 0.68 | 1.66 | 0.794 |  |
| **Educational attainment (Ref None)** | -- |  |  |  |  |  |  |  |  |
| High School/ GED | 1.48 | 0.78 | 2.81 | 0.229 | 1.48 | 0.87 | 2.53 | 0.149 |  |
| College / postgraduate | 0.74 | 0.36 | 1.53 | 0.419 | 0.93 | 0.47 | 1.84 | 0.840 |  |
| **Neighbourhood safety (Ref Good/excellent)** | -- |  |  |  |  |  |  |  |  |
| Fair/Poor | **0.49** | **0.25** | **0.94** | **0.032** | **0.43** | **0.20** | **0.96** | **0.040** |  |
| **Employment status (Ref Employed)** | -- |  |  |  |  |  |  |  |  |
| Unemployed/ Inactive | 0.98 | 0.55 | 1.77 | 0.952 | 1.36 | 0.51 | 3.63 | 0.544 |  |
| Retired | 0.90 | 0.55 | 1.49 | 0.691 | 0.70 | 0.32 | 1.54 | 0.376 |  |
| **Wealth, quartiled (Ref Quartile 1)** | -- |  |  |  |  |  |  |  |  |
| Quartile 2 | 0.86 | 0.48 | 1.54 | 0.612 | 1.02 | 0.51 | 2.04 | 0.966 |  |
| Quartile 3 | 1.00 | 0.52 | 1.92 | 0.994 | 1.14 | 0.56 | 2.30 | 0.720 |  |
| Quartile 4 | 0.63 | 0.31 | 1.26 | 0.189 | 0.84 | 0.39 | 1.78 | 0.644 |  |
| **Satisfied with aging (Ref Yes)** | -- |  |  |  |  |  |  |  |  |
| No | 0.63 | 0.29 | 1.35 | 0.232 | 0.48 | 0.21 | 1.14 | 0.094 |  |
| **Satisfied with Life (Ref Yes)** | -- |  |  |  |  |  |  |  |  |
| No | 1.16 | 0.59 | 2.31 | 0.662 | 1.05 | 0.55 | 2.01 | 0.880 |  |
| **See friends (Ref Yearly/less)** | -- |  |  |  |  |  |  |  |  |
| Monthly | 0.91 | 0.43 | 1.92 | 0.797 | 1.02 | 0.43 | 2.40 | 0.972 |  |
| Weekly | 0.98 | 0.42 | 2.25 | 0.958 | 0.97 | 0.37 | 2.55 | 0.944 |  |
| **Attend religious services (Ref Yearly/Less)** | -- |  |  |  |  |  |  |  |  |
| Monthly | **2.17** | **1.31** | **3.62** | **0.003** | 0.84 | 0.48 | 1.45 | 0.524 |  |
| Weekly | **1.95** | **1.15** | **3.30** | **0.013** | 0.61 | 0.36 | 1.03 | 0.063 |  |
| **Depression CES-D (Ref None)** | -- |  |  |  |  |  |  |  |  |
| Present | 1.49 | 0.81 | 2.74 | 0.198 | 1.06 | 0.54 | 2.06 | 0.868 |  |
| **Smoker** | -- |  |  |  |  |  |  |  |  |
| Yes | 1.67 | 0.93 | 3.01 | 0.088 | 2.08 | 0.86 | 5.02 | 0.104 |  |
| **Self-rated health (Ref Good/excellent)** | -- |  |  |  |  |  |  |  |  |
| Fair/Poor | 0.63 | 0.35 | 1.13 | 0.119 | 0.90 | 0.53 | 1.53 | 0.695 |  |
| **iADL (Ref None)** | -- |  |  |  |  |  |  |  |  |
| Difficulties with activities | 0.78 | 0.42 | 1.43 | 0.420 | 0.88 | 0.53 | 1.46 | 0.618 |  |
| Unable to do activities | **0.21** | **0.05** | **0.83** | **0.026** | **0.16** | **0.04** | **0.77** | **0.022** |  |
| **Long term conditions (Ref None)** | -- |  |  |  |  |  |  |  |  |
| Yes | 1.10 | 0.72 | 1.67 | 0.661 | 1.57 | 0.96 | 2.55 | 0.070 |  |
| **Total cognition score, quartiled (Ref Quartile 1)** | -- |  |  |  |  |  |  |  |  |
| Quartile 2 | 1.28 | 0.68 | 2.42 | 0.447 | 1.31 | 0.80 | 2.15 | 0.288 |  |
| Quartile 3 | 0.58 | 0.29 | 1.15 | 0.118 | 0.70 | 0.38 | 1.28 | 0.242 |  |
| Quartile 4 | 0.76 | 0.38 | 1.52 | 0.437 | 0.73 | 0.36 | 1.47 | 0.380 |  |
| **Arts index** | 0.77 | 0.57 | 1.05 | 0.098 | 0.78 | 0.58 | 1.06 | 0.111 |  |

Note. Dashes indicate reference category.

Difficulties participating in the arts is: neutral/disagree vs agree to 'it is difficult for me to participate in the arts'

Columns A-B show age stratified analyses
