# Supplementary material for: Difference in predictors and barriers to arts and cultural engagement with age in the United States: A cross-sectional analysis using the Health and Retirement Study
Source: PLoS One. 2021 Dec 20;16(12):e0261532. doi: 10.1371/journal.pone.0261532 (PMC8687585; doi:10.1371/journal.pone.0261532)
Supplement: Attachment — Submitted filename: Reviewer response letter_EP.docx [file pone.0261532.s004.docx]

*We would like to thank the editors and reviewers for the opportunity to revise and resubmit, and for the helpful comments which have strengthen our manuscript. We have responded to each comment point-by-point below.*

**Reviewer #1**: This manuscript deals with a topic that is quite relevant in the current context.
The introduction is well written, and I recommend that the authors insert the study's hypotheses at the end of the introduction. I also suggest that they provide a more robust justification for doing this work.

*Many thanks for the kind comments and are pleased you found this topic relevant and the writing clear.*

*We have now added a sentence to more clearly introduce our study justifications, which are further outlined in paragraphs two and three of the introduction section. These justifications centre around the observation that while we know there are predictors of and barriers to arts and cultural engagement- there is little research on how these factors change as people age. Further, there has been little research into engagement versus wanting to engage (but being unable to do so) which this paper also examines. Further, we add to existing literature by examining predictors and barriers for different age groups of older adults, as well as active rather than passive arts engagement.*

*We have also expanded the hypotheses to make this clearer.*

Regarding the method, I recommend that the authors insert the inclusion and exclusion criteria of the participants and the instruments used to identify the constructs.

*We have now included a statement in the methods to make clearer our process for selecting sample participants. This statement clearly states the inclusion criteria as well as a numeric breakdown of sample selection. If any validated instruments were used in the study (for example the CES- D or iADL), they were appropriately referenced. Therefore, the remaining variables were taken from questions which were available to us in the HRS survey and are summarised in the methods section.*

The data analysis should also be better described, presenting all the statistical procedures used in work. I recommend that the authors make a detailed description of the tables throughout the text.

*We have now updated the statistical analysis section and added text under each table to further*

*clarify the statistical procedures used.*

The Discussion needs to be deepened, comparing the results found in this study with others in the literature.

*We have now expanded upon the discussion with additional references and comparisons to previous literature, relevance of the findings, and suggested policy implications.*

**Reviewer #2**: In this article, the authors provided interesting insights to predictors and barriers to participation and engagement with art and how these may change with aging. As a researcher who focuses on the basic research of aging, it was great to read and learn about more wholistic contributors to the aging process and about potential impediments to aging individuals to take advantage of available option that can affect healthy aging. The article is well-written and thought provoking. However, there are a few minor adjustments that the authors should consider prior to publishing the article.

1. As PLOS ONE readers come from diverse backgrounds and areas of expertise, the authors should justify and explain the reasons for collapsing the responses which results in binary data (under methods -> Outcome variables)

*We have now noted in our description of the outcome variables of the methods section that small sample size was the reason for our collapsing into binary categories.*

2. The first discussion paragraph is simply a restatement of the results and does not add to the discussion. That is also somewhat true for the second discussion paragraph as well. In fact, the first sentence that provides critical assessment of the data comes only in the 7th sentence of the 3rd discussion paragraph. The authors should consider rewriting some of the discussion section to reflect a stronger critical assessment of the data and its relevancy to everyday life and health outcomes of aging individuals.

*We agree with the reviewers that a large portion of the discussion is focused on restating the results. However, as this study examined a large number of predictors and barriers to engagement across several outcomes, it is important these are summarised for our readers. To aid readability, we have now disaggregated these summaries and added subheadings. We have also extended the comparisons to previous literature and added text on the relevancy to life and health of ageing people.*

Other than these two comments, I think that this short manuscript is a great addition to PLOS ONE and that many readers may find it useful.

*Thank you for your kind comments about the manuscript, and we believe the new additions have further strengthened it.*
